# Study on MMA and BA Emulsion Copolymerization Using 2,4-Diphenyl-4-methyl-1-pentene as the Irreversible Addition–Fragmentation Chain Transfer Agent

**DOI:** 10.3390/polym12010080

**Published:** 2020-01-02

**Authors:** Zuxin Zhang, Daihui Zhang, Gaowei Fu, Chunpeng Wang, Fuxiang Chu, Riqing Chen

**Affiliations:** Institute of Chemical Industry of Forest Products, Chinese Academy of Forestry; National Engineering Laboratory for Biomass Chemical Utilization; Key Laboratory of Chemical Engineering of Forest Products, National Forestry and Grassland Administration; Key Laboratory of Biomass Energy and Material, Nanjing 210042, China; zinc2019@163.com (Z.Z.); zdh0824@163.com (D.Z.); 15638863071@163.com (G.F.); wangcpg@163.com (C.W.); chufuxiang@caf.ac.cn (F.C.)

**Keywords:** 2,4-diphenyl-4-methyl-1-pentene, irreversible addition–fragmentation chain transfer, emulsion polymerization, MMA, BA

## Abstract

As a chain transfer agent, 2,4-diphenyl-4-methyl-1-pentene (αMSD) was first introduced in the emulsion binary copolymerization of methyl methacrylate (MMA) and butyl acrylate (BA) based on an irreversible addition–fragmentation chain transfer (AFCT) mechanism. The effects of αMSD on molecular weight and its distribution, the degree of polymerization, polymerization rate, monomer conversion, particle size, and tensile properties of the formed latexes were systematically investigated. Its potential chain transfer mechanism was also explored according to the ^1^H NMR analysis. The results showed that the increase in the content of αMSD could lead to a decline in molecular weight, its distribution, and the degree of polymerization. The mass percentage of MMA in the synthesized polymers was also improved as the amounts of αMSD increased. The chain transfer coefficients of αMSD for MMA and BA were 0.62 and 0.47, respectively. The regulation mechanism of αMSD in the emulsion polymerization of acrylates was found to be consistent with Yasummasa’s theory. Additionally, monomer conversion decreased greatly to 47.3% when the concentration of αMSD was higher than 1 wt% due to the extremely low polymerization rate. Moreover, the polymerization rate was also decreased probably due to the desorption and lower reactivity of the regenerative radicals from αMSD. Finally, the tensile properties of the resulting polyacrylate films were significantly affected due to the presence of αMSD.

## 1. Introduction

Due to the growing environmental concerns, emulsion polymerization has become a vital method for polymer production with its environmentally friendly process, high polymerization rate, high monomer conversion, and excellent heat dissipation performance [1]. Many engineering efforts have also been put on the multiscale modeling of this process [2,3]. Chain transfer agent (CTA) as an important component, including reversible addition–fragmentation chain transfer (RAFT) and traditional CTAs, have been widely employed to control the molecular weights of synthesized polymers during the emulsion polymerization. Although many works about RAFT CTAs have recently been conducted, the complex processes and strict operation conditions of RAFT CTAs limit their wide applications. [4,5,6] For traditional CTAs, including mercaptans [7,8,9,10], alcohols [11], and halides [12], several disadvantages have also been observed, such as an unpleasant smell for mercaptans and serious environmental pollution for halides. In the case of alcohols, the amount used as a molecular weight regulator in the polymerization is typically large (10%–20% relative to monomer mass), and it is also not considered to be an environmentally friendly agent.

**Scheme 1:**
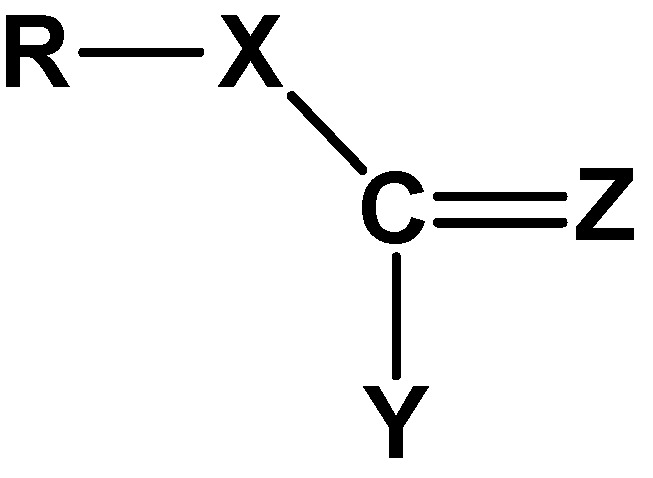
General chemical structure of the irreversible addition–fragmentation chain transfer (AFCT) agent.

An irreversible addition–fragmentation chain transfer (AFCT) agent has been developed to solve the above problems. It displayed higher chain transfer efficiency, lower toxicity, and lower dosage. The first report about the AFCT agent was presented in 1988, when Meijs et al. [13] used α-benzyl-oxy-styrene as a chain transfer agent for the preparation of poly(methyl methacrylate) and polystyrene with low molecular weights. Then, various new types of AFCT agents were reported in research papers [14,15,16,17,18,19,20,21] and patents [22,23,24]. The common structure of the irreversible AFCT agent is shown in Scheme 1. C=Z is a double bond that is vulnerable to be attacked by the chain radical. R-X is a single bond that can be broken easily, and Y is a group to activate the reaction. The general mechanism for the irreversible AFCT agent mainly contains three steps. Firstly, the chain radical is added to the unsaturated double bond of C=Z. Then, the addition product offers a leaving group radical (R•) by the decomposition. Eventually, the novel radical reinitiates the polymerization process.

**Scheme 2:**
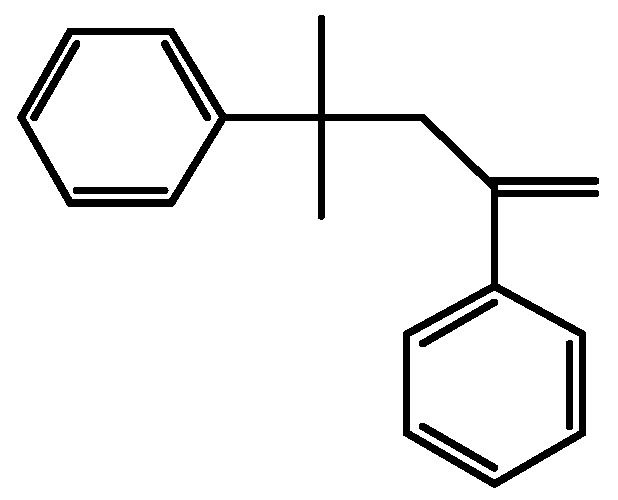
Chemical structure of 2,4-diphenyl-4-methyl-1-pentene (αMSD).

2,4-Diphenyl-4-methyl-1-pentene (αMSD), shown in Scheme 2, is a typical irreversible AFCT which can be prepared by α-methylstyrene dimerization in the presence of an aqueous sulfuric acid catalyst in the liquid–liquid mode of operation [25,26].

**Scheme 3:**
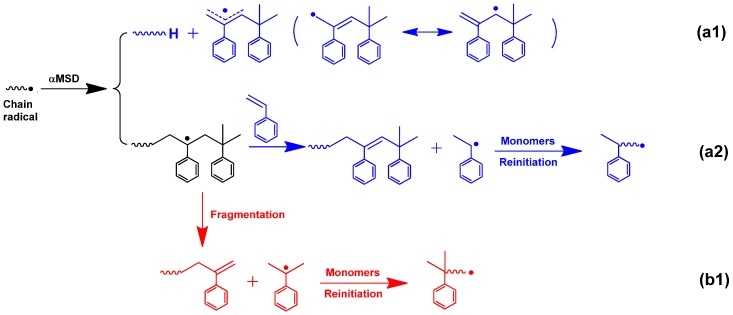
The two chain transfer mechanisms of αMSD in the polymerization of styrene. Mechanism of alkyl hydrogen suggested by Fisher et al.: transferring to chain radical (**a1**) and monomers (**a2**). (**b1**) Addition–fragmentation chain transfer mechanism reported by Yasummasa et al.

Currently, αMSD has been introduced into solution and bulk radical polymerization in order to synthesize polystyrene with decreased molecular weights and narrowed molecular weight distribution [27,28]. For the specific regulation mechanism of αMSD in the polymerization (Scheme 3) Fisher et al. [29] proposed that the chain transfer would proceed in two cases. In the first case, alkyl hydrogen carrying a free electron abstracted from an αMSD was combined with a chain radical to form a dead chain (Scheme 3a1), whereas the residual structure of αMSD carrying a free electron could reinitiate the polymerization process (Scheme 3a1). In the second case, a chain radical was directly added to αMSD and then formed a transient state radical. An alkyl hydrogen radical divided from the αMSD structure of the latter radical was then transferred to a monomer to form a new radical. Afterwards, the new radical was able to reinitiate the polymerization. Finally, the residual part of the transient state radical became a dead chain, and the end group of the dead chain was an internal olefin (Scheme 3a2). However, according to the results of the thermal polymerization of styrene, Yasummasa et al. [30] concluded that the polymer chain radical would first be added to the terminal double bond of αMSD to produce an intermediate radical, and then this radical would decompose into a polymer chain with a terminal double bond and a cumyl radical. Finally, the cumyl radical would reinitiate the polymerization (Scheme 3b1) [31,32,33,34]. Furthermore, the regulation mechanism study of αMSD in the radical bulk and suspension polymerization of styrene has been observed to support Yasummasa’s theory.

Although there have been some developments for the radical bulk and suspension polymerization using αMSD, its utilization in the emulsion polymerization has never been reported. In the present study, different levels of αMSD were incorporated into poly-(meth) acrylate emulsion via the semi-continuous emulsion copolymerization of MMA and BA to investigate its effects on the properties of the resulting polymer emulsion, such as molecular mass, polymerization rate, and particle size. The αMSD regulation mechanism was also discussed. 

## 2. Materials and Methods

Reagents: αMSD (Shanghai Dibo Chemical Technology, Shanghai, China), MMA and BA (monomers), nonylphenol polyoxyethylene ether (NP-10, nonionic emulsifier), alkyl phenol ether sulfosuccinate sodium salt (OS, nonionic emulsifier), sodium dodecyl sulfate (SDS, anionic emulsifier), disodium hydrogen phosphate dodecahydrate (Na_2_HPO_4_·12H_2_O, stabilizer, Sinopharm Chemical Reagent, Shanghai, China), and ammonium persulfate (APS, initiator, Nanjing Reagent, Nanjing, China) were used as received. Distilled deionized water (DDW) was used as the reaction medium.

Emulsion polymerization: The emulsion polymerization of (meth) acrylates was carried out in a 500 mL 4-neck glass reactor equipped with a mechanical stirrer, a reflux condenser, a nitrogen gas inlet, and a thermometer. These following substances and amounts were mixed into the DDW (158.40 g): SDS (2.60 g), NP-10 (0.65 g), OS (0.65 g), and Na_2_HPO_4_·12H_2_O (0.40 g), followed by mechanical stirring to form an aqueous solution. The (meth) acrylate monomers MMA (65 g) and BA (65 g) were homogeneously mixed with various amounts of αMSD under a mechanical stirring. The formed organic phase was then added dropwise into the aqueous solution to form an emulsion solution. A total of 10 g of the resulting solution and 0.55 g of initiator solution (0.05 g APS in 0.50 g DDW) were poured into the glass reactor and then heated to 75 °C. Finally, a residual pre-emulsion and 6.6 g of initiator solution containing 0.60 g APS were added dropwise simultaneously into the reactor to polymerize at 75 °C for 4 h under a nitrogen atmosphere.

Characterization: Monomer conversion was determined by a gravimetric analysis of samples withdrawn from the glass reactor during polymerization, and these samples were quenched and dried at 120 °C for 2 h. The conversion calculations were based on the total amount of solid of each emulsion sample taken at the beginning of the polymerization. The number and weight-average molecular weights of the polymers were obtained by the gel permeation chromatography (GPC) via a Malvern Viscotek3580 system, which was calibrated using standard narrow molecular weight polystyrene samples. The dried polymer films were dissolved in GR-grade tetrahydrofuran eluent and filtered through a polytetrafluoroethylene (PTFE) filter (0.22 μm) before the injection. Analysis was conducted with a flow rate of 1 mL per minute at 40 °C. Average particle size (*d*_p_, light intensity-weighted) and particle-size distribution index (PDI) were measured by a laser light-scattering method using a Malvern ZETASIZER Nano at 25 °C. All latex samples for the measurement were diluted 50 times with DDW. The PDI was a dimensionless coefficient denoting the broadness of the distribution diameter, and it was produced by a second-order method of cumulant analysis [35].

The ^1^H NMR spectra of the resultant polymers were measured with a Bruker DMX 300 NMR. The polymer samples were dissolved into CDCl_3_ (10–15 wt/vol %) at 25 °C. The tensile properties of the dried polymer films were measured on electronic tensile machine CMT7504 with a crosshead speed of 50 mm/min and a load cell of 250 N at room temperature. The stress–strain curves of the polymer films were plotted to estimate the films’ strength and toughness variations.

## 3. Results and Discussion

### 3.1. Effect of αMSD on the Physichemical Properties of Polymers

Different levels of αMSD were introduced into the emulsion polymerization of (meth) acrylate in order to effectively regulate the physical properties of the resulting polymers. Its effect on average molecular mass, polymerization degree, and monomer conversion is shown in Table 1.

As the addition of αMSD increased from 0% to 0.1%, the number-average molecular weights were evidently decreased from 40.56 × 10^4^ to 28.58 × 10^4^ g/mol, whereas monomer conversion underwent a slight decrease to 97.8%. When αMSD concentration increased from 0.1% to 1%, both molecular weights and monomer conversion were significantly decreased, as the presence of αMSD was able to effectively interfere with the polymerization process. In terms of the dispersity (*Ð*) of the molecular weight, the addition of αMSD contributed to the gradual decrease of the dispersity to nearly 1.60 due to a mathematical artefact in the definition of dispersity [36]. However, despite its efficiency in adjusting molecular weights and *Ð*, only 44.6% monomer conversion was accomplished when the concentration of αMSD was 3%. These results indicate that the utilization of αMSD as a chain transfer agent is effective in the adjustment of the molecular weights and the dispersity *Ð* of synthesized polymers, but a further addition of αMSD might compromise the monomer conversion.

**Figure 1:**
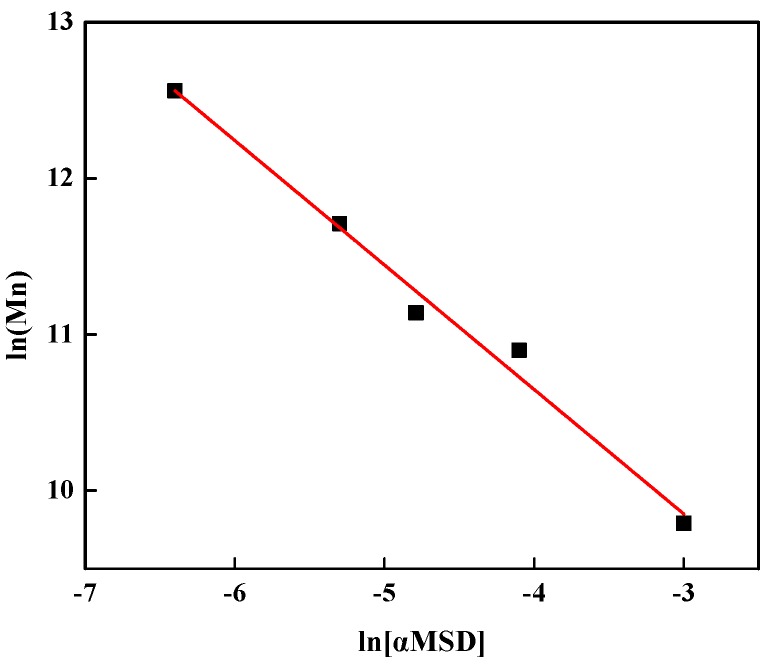
A log–log curve of M_n_ and αMSD concentration.

Figure 1 was obtained by plotting the change in logarithm of the concentration of αMSD (ln [αMSD]) as a function of the logarithm of *M*_n_ (ln [*M*_n_]). It showed that the influence of αMSD concentration on M_n_ was estimated to be *M*_n_∝[αMSD]^−0.797^, and the fitting linear expression was *y* = 7.458 − 0.797*x* (*R-Square* = 0.983, *y*-ln [*M*_n_], *x*-ln [αMSD]). The fitting result quantitatively described the effect of αMD on molecular weight of synthesized polymers, which could be used to estimate the appropriate CTA dosage according to different mass requirements.

In addition, ${\bar{X}}_{n}$ in Table 1 was obtained from Equation (1) [37]. As MMA was expected to polymerize first, followed by the polymerization of BA [38], Equation (1) is shown as follows.

(1) $${\bar{X}}_{n}=M_{n}\left(\frac{x}{m_{1}}+\frac{1-x}{m_{2}}\right),$$

(2) $$F_{1}=\frac{f_{1}{}^{0}-\left(1-C\right)f_{1}}{C}$$

(3) $$C=1-{\left(\frac{f_{1}}{f_{1}{}^{0}}\right)}^{\alpha }{\left(\frac{f_{2}}{f_{2}{}^{0}}\right)}^{\beta }{\left(\frac{f_{1}{}^{0}-\delta }{f_{1}-\delta }\right)}^{\gamma }$$

where *x* is the mass fraction of MMA in the synthesized copolymers; *F*_1_ as the mole fraction of MMA in the copolymer is calculated by Meyer’s [37] copolymerization equations (Equations (2) and (3)); *m*_1_ and *m*_2_ are the relative molecular masses of MMA and BA, respectively; *f*_1_^0^ and *f*_1_ are the mole fractions of MMA in the monomer phase at the beginning and after a certain conversion of the polymerization, respectively; *f*_2_^0^ and *f*_2_ are the mole fractions of BA at different stages; *C* is a monomer conversion; *α*, *β*, *γ*, and *δ* are coefficients relating to the properties of monomers, which are shown in Table 2. 

**Figure 2:**
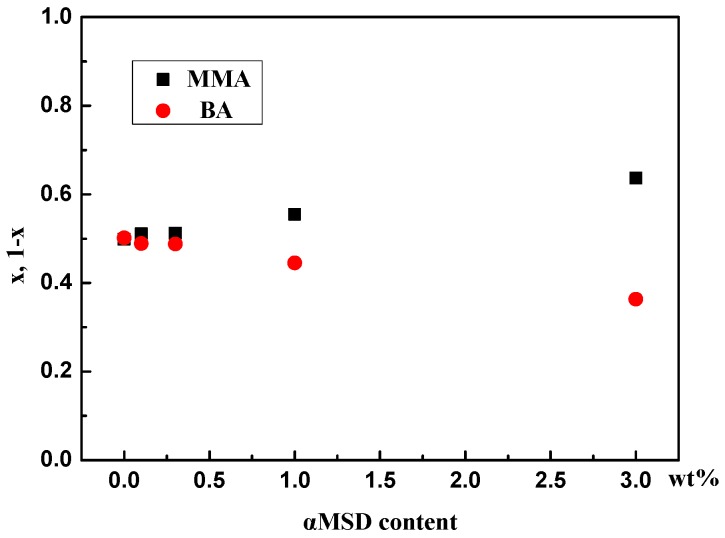
Variation tendencies of the average quality proportion of MMA (*x*) and BA (1-*x*) in a polymer in the presence of αMSD.

The relative variation tendencies of *x* and 1-*x* could be seen in Figure 2. As the amounts of initiator and αMSD in the polymerization process were very small, their proportions in the polymer were ignored. It is evident that x increased gradually with increasing amounts of αMSD. This indicated that αMSD affected not only the molecular weights of polymers, but also its composition.

To analyze the potential reason, the reactivity ratios were calculated according to a semi-empirical formula, equation *Q*-*e* (Equations (4) and (5)).

(4) $$r_{a}=\frac{Q_{a}}{Q_{b}}\exp[-e_{a}\left(e_{a}-e_{b}\right)],$$

(5) $$r_{b}=\frac{Q_{b}}{Q_{a}}\exp[-e_{b}\left(e_{b}-e_{a}\right)]$$

where *Q* and *e* represent the conjugation degree and polarity of monomer substituents, respectively. *r_a_* and *r_b_* are the reactivity ratios of monomers *a* and *b*, respectively. In the case of *r_a_*, a higher value means a larger self-polymerization trend. Alternatively, a lower value indicates a larger copolymerization trend.

During the calculation, the *Q* and *e* values for αMSD were estimated using *Q* and *e* of alpha methyl styrene (AMS), as αMSD was a dimer of AMS. The specific *Q* and *e* values of AMS, MMA, and BA were listed in Table 3, and the calculated reactivity ratios of the three monomers were given in Table 4.

Where *r*_1_ and *r*_3_ are the reactivity ratios for MMA to BA and AMS; *r*_2_ and *r*_6_ are the reactivity ratios for BA to MMA and AMS; and *r*_4_ and *r*_5_ are the reactivity ratios for AMS to MMA and BA, respectively.

As given in Table 4, *r*_1_ > *r*_2_ revealed that MMA had a greater polymerization activity than BA, which contributed to a higher *x* (>0.5) in the early polymerization period. For the sample without αMSD addition (Run 1), BA’s mass fraction in the polymers gradually increased up to 50% in the later polymerization period. However, this process would be terminated earlier in the presence of αMSD. Because of the chain transfer reaction of αMSD, the propagation process was terminated significantly earlier with an increase in the amount of αMSD. As a result, the growth of BA’s mass fraction in the polymer was terminated earlier and earlier. Finally, MMA’s mass fraction in the polymer increased, whereas the mass fraction of BA was lowered with an increase in the amount of αMSD.

In addition, *r*_1_ > *r*_3_ indicated that MMA had a greater tendency to copolymerize with AMS than BA. Similarly, *r*_2_ > *r*_6_ indicated that BA tended to copolymerize with AMS rather than MMA. In other words, both MMA and BA were more likely to copolymerize with AMS (αMSD). This could indirectly explain why αMSD effectively controlled the molecular weights of the polymer. For AMS, *r*_4_ ≈ *r*_5_, there was an almost equal possibility of AMS to copolymerize with MMA or BA.

To compare the regulation reactivity differences of αMSD as a chain transfer agent to MMA and BA more accurately, its transfer coefficients (*C*_tr_) to each single monomer were calculated with the following expression (Equation (6)). It was derived by Alfrey [39] for the case of binary copolymerization in the presence of small amounts of CTA, based on the assumption that the only effect of the chain transfer reaction was to terminate one growing molecular chain and start another, i.e., ignoring cases in which a chain transfer also leaded to the termination of the kinetic chain.

(6) $$K=AC_{\mathrm{tr},a}\frac{\left[A]+[B\right]}{\left[A]+r_{a}[B\right]}+BC_{\mathrm{tr},b}\frac{\left[A]+[B\right]}{r_{b}[A]+[B]},$$

where *A* and *B* are mole fractions of monomer A and monomer B, respectively, in the polymer; [*A*] and [*B*] are the mole fractions of monomer A and monomer B in the whole monomers; *r_a_* and *r_b_* are the reactivity of the two monomers; and *K* is the slope of a Mayo-like plot (i.e., 1/${\bar{X}}_{n}$ vs. total monomer concentration, Equation (7)) [40,41].

(7) $$\frac{1}{{\bar{X}}_{n}}=\frac{1}{{\bar{X}}_{n,0}}+K\frac{\left[C\right]}{\left[M\right]},$$

where [*C*] and [*M*] are concentrations of the CTA and whole monomers, respectively; ${\bar{X}}_{n}$ is the average degree of polymerization for the polymer at a given [*C*]; and ${\bar{X}}_{n,0}$ is for the polymer in the absence of CTA.

In this work, MMA and BA were regarded as monomers A and B, respectively, as αMSD was used as the CTA. On the basis of the above analysis, the values of the individual transfer coefficients (C_tr,a_ and C_tr,b_) for a particular monomer ratio were successfully obtained by the two simultaneous equations (Equations (6) and (7)).

**Figure 3:**
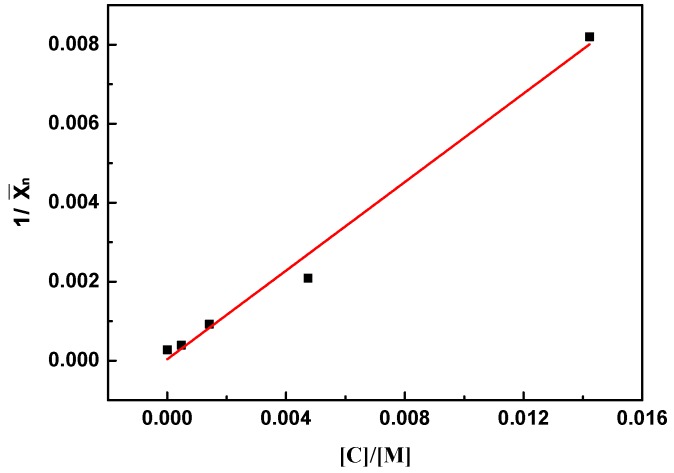
Mayo plot of 1/${\bar{X}}_{n}$ vs. total monomer concentration for MMA–BA copolymerization in the presence of αMSD.

Figure 3 displayed the fitting result of the Mayo plot, and *K* was derived from the slope of the plot: 0.56 (R-Square = 0.986). *K* remained unchanged as long as the monomer ratio of MMA and BA was a constant. As a consequence, *C*_tr,a_ and *C*_tr,b_ could be calculated through Equation (6) by choosing any two runs (including αMSD) in Table 1, such as Run 2 and Run 4, and the result was shown in Table 5.

If this polymerization system was treated as a terpolymerization of MMA, BA, and αMSD, the two *C*_tr_s of αMSD could be calculated by the reactivity ratios of the three monomers on the basis of *Q*-*e* values. According to the definition of *C*_tr_, the *C*_tr_s values of αMSD should be kinetically equivalent to the reciprocals of the reactivity ratios of any two monomers. The calculation results were shown in Table 5.

As shown above, the *C*_tr_s values of αMSD determined by different methods were obviously different. *C*_tr,MMA_ and *C*_tr,BA_ were 0.62 and 0.47 according to Alfrey’s theory, respectively. However, when using *Q*-*e* method, the former was 2.12 and the latter was increased to 14.28. The difference could be explained from two aspects: First, the *C*_tr_ calculations based on the *Q*-*e* method utilized AMS’s reactivity ratios to represent αMSD’s. Nevertheless, as a dimer of AMS, αMSD’s structure was more complex than AMS, and the former had more steric effects when it reacted with other monomers. Thus, their reactivity ratios as well as *C*_tr_s should be different. Second, there were some inherent errors in calculating the reactivity ratios using the *Q-e* method.

In contrast to the *Q-e* method, the substitution of AMS for αMSD was avoided in Alfrey’s theory, so the *C*_tr_s values of αMSD should be more accurate. In addition, the almost same values of *C*_tr,MMA_ and *C*_tr,BA_ indicated that αMSD has almost same chain transfer possibilities with both MMA and BA, which was consistent with the above analysis of reactivity ratios in Table 4.

**Figure 4:**
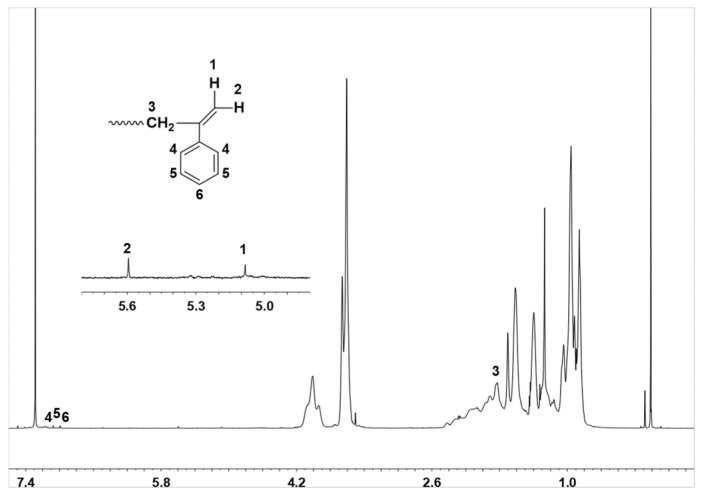
^1^HNMR spectrum of the resulting polymer in the presence of αMSD (content: 1%).

**Scheme 4:**
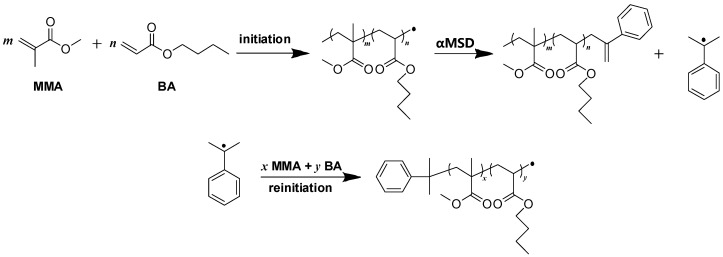
Chain transfer mechanism of αMSD in MMA and BA emulsion copolymerization.

As described above, few studies have been conducted to use αMSD as a chain transfer agent in the emulsion polymerization. Therefore, we have used NMR analysis to reveal its potential mechanism. In the polymerization process, chain propagation would be terminated by the irreversible AFCT agent. Therefore, a part of the polymer’s terminal structures consists of the AFCT agent. On this basis, the mechanism for αMSD could be revealed by a polymer terminal structure analysis. The ^1^H NMR analysis of the resulting polymer using 1% αMSD showed that two signals of similar intensities at approximately 5.0, 5.1, and 5.6 ppm were observed as the characteristic peaks of the two syntonic hydrogen atoms of the terminal double bonds (Figure 4). Moreover, multiple signals at ~7.0 ppm were present, which were ascribed to the substituted benzene ring from the αMSD. Accordingly, these results confirmed that emulsion polymerization of acrylates using αMSD as a chain transfer agent followed Yasummasa’s mechanism. The detailed regulation mechanism is illustrated in Scheme 4.

### 3.2. Effect of αMSD on the Polymerization Rate

**Figure 5:**
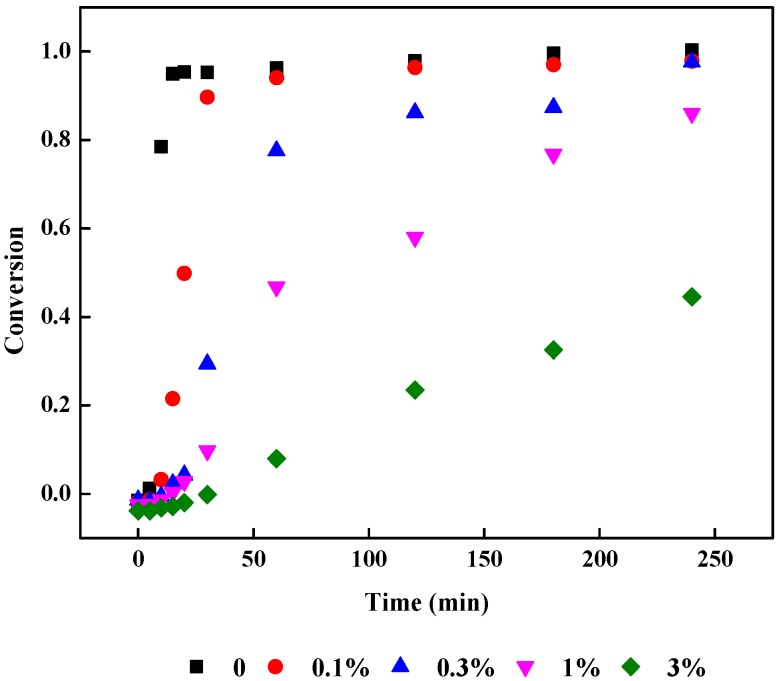
Monomer conversion as a function of reaction time at various molar ratios between αMSD and acrylate monomers (see Table 1).

**Figure 6:**
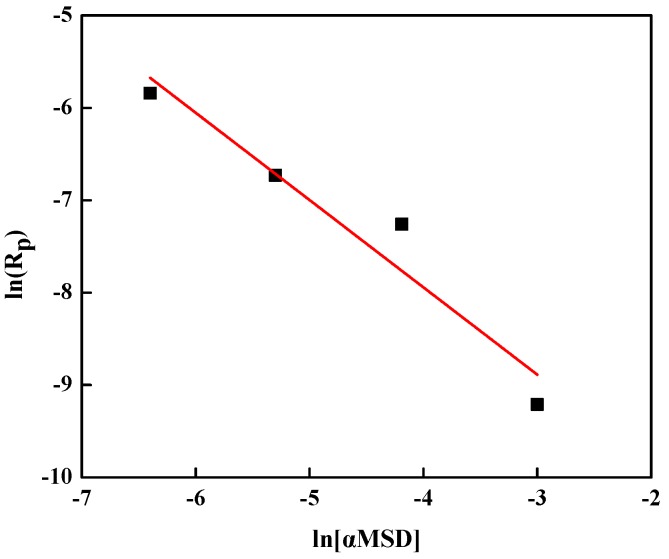
Rate of polymerization as a function of various αMSD concentrations. Slope of linear fit = −0.945 (*R-Square* = 0.905).

As αMSD has an obvious influence on polymer molecular weights, it was necessary to study if its presence would also affect the polymerization rate (*R_p_*). In the emulsion polymerization, the constant speed stage was typically used as the indicator of total polymerization rate, and it could be calculated from the conversion–time curve. Figure 5 showed the monomer conversion versus reaction time in the presence of αMSD at various concentrations. The slopes of the curves directly reflected *R_p_*. It was clear that the presence of αMSD could decrease the *R_p_* as compared to samples without αMSD. Moreover, as the increase in the quantities of αMSD, *R_p_* was decreased more significantly. For example, the monomer conversion only reached 0.4 after 250 min when 3% of αMSD was added, whereas its conversion approached 100% without αMSD addition. Furthermore, the effect of αMSD concentration ([αMSD]) on *R_p_* was further correlated via constructing a log–log plot of *R_p_* and [αMSD], as shown in Figure 6. As expected, the result was quantitatively estimated to be *R_p_*∝[αMSD]^−0.945^ (R-Square = 0.905).

The potential reasons for the decrease in *R_p_* in the presence of αMSD may be explained from two aspects. First of all, the overall rate constant of the scission process of the leaving group may be lower than propagation [20]. The efficient transfer reaction of an irreversible AFCT agent required that transitional radicals, created by the addition reaction of the irreversible AFCT agent and chains radicals, could easily fragment to generate new radicals to reinitiate the polymerization process. As a result, retardation would happen. In fact, the novel radical (isopropyl benzene radical, seen in Scheme 4) in this paper, had less access to reinitiate polymerization than the primary radical (sulfate radical anion) due to its steric hindrance. Thus, the macroscopic reaction rate decreased. Second, radicals derived from both the initiator and leaving groups might escape from micelles or latex particles (desorption) and then be terminated with radicals in either the water phase or polymer particle (reabsorption). This desorption phenomenon had been successfully verified in the emulsion polymerization, and the related theoretical model had been previously established [42,43,44]. The sulfate radical anion is more hydrophilic than the isopropyl benzene radical. As a result, the desorption number of the former was much greater than latter, and the latter tended to stay in the original particles. This led to serious loss of the high-active free radicals, and in addition, the remaining free radicals in the latex particles were not active enough, which further exacerbated the decline in *R_p_*.

### 3.3. Effect of αMSD Particle Size and Relevant Parameters

The influence of αMSD concentration on latex particle size(*d_p_*), its distribution (*PDI*), and particle number (*N*_c_) were also explored, and the results were shown in Table 6. The particle number of the resulting emulsion was calculated using an Equation (8).

(8) $$N_{c}=\frac{6m}{\pi \rho {d_{p}}^{3}}\times 10^{21},$$

where *m* is the solid content of the dispersed phase (containing monomer and polymer at a given conversion and expressed in g/mL^−1^), and *ρ* represents the particle density.

As shown in Table 6, *d*_p_ declined from 109.38 to 46.88 nm as αMSD dosage was varied from 0% to 3%, whereas *N*_c_ was enhanced from 1.67 × 10^17^ to 20.79 × 10^17^ mL^−1^. Moreover, *PDI* started to dramatically increase when the αMSD dosage was higher than 1%.

According to recent reports [45,46], the exit and entry rates of radicals and monomers into particles have strong effects on miniemulsion polymerization kinetics. In this study, isopropyl benzene radicals derived from αMSD increased the whole radical concentration in the particles, which contributed to the greater desorption of sulfate radical anions. Thus, these sulfate radical anions were resorbed by other micelles, and more micelles could be transformed into polymer particles. This explained the increase in *N_c_* and the decrease in *d_p_*. Generally speaking, the increase in *N*_c_ should be accompanied by inhibition of the termination reaction, leading to a higher reaction rate. However, the actual *R_p_* was decreased. In fact, actual variations of *N*_c_ and *d_p_* were not necessarily related to *R_p_*. A mass transfer resistance occurred during the transfer process of sulfate radical anions among the micelles, water, and particles. This would counteract the slight increase in *R_p_* caused by the lower degree of termination. As a result, the variations of *N*_c_ and *d_p_* failed to improve *R_p_*.

### 3.4. The Effect of αMSD on Tensile Properties of Polymer Films

**Figure 7:**
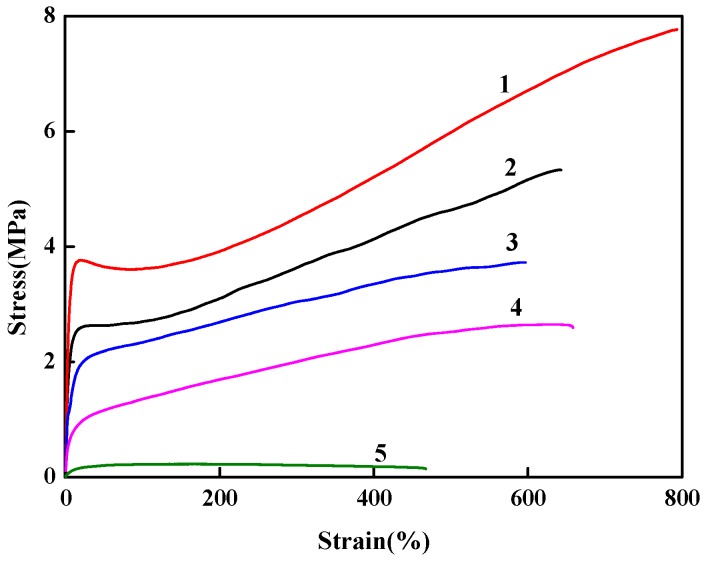
Stress–strain curves of latex films with different αMSD concentrations.

The tensile properties (*E*, *σ*_p_, and *ε*_b_) of the latex films prepared from polyacrylate emulsion at various αMSD concentrations were determined, and the stress–strain curves were shown in Figure 7. The results were summarized in Table 7. As expected, αMSD addition had an impact on the mechanical properties of the latex film. With the increase in αMSD from 0% to 1%, *E* and *σ*_p_ of the resultant films were reduced by nearly 75% and 50%, respectively, whereas *ε*_b_ was only slightly affected. Furthermore, the obvious yield point of the resultant polymers gradually became less obvious when the amount of αMSD was above 0.3%, indicating that the polymer films changed from hard to soft [47]. When the amount of αMSD reached to 3%, *E* and *ε*_b_ were only 2.00 and 0.23 MPa, respectively, and *ε*_b_ had decreased to 467%. The toughness of the resultant films was significantly decreased, and the film was soft and weak. The decrease in the molecular weights of polymers was the main reason to lead to the poor mechanical properties.

## 4. Conclusions

In this study, it was shown that αMSD was a highly effective CTA to control the molecular weights in MMA and BA emulsion copolymerization. Molecular masses and their distributions, as well as the mass proportion of MMA in the polymers, were gradually decreased when increasing the dosage of αMSD. Meanwhile, the final monomer conversion was also declined as the αMSD concentration increased above 1%, indicating its apparent inhibition ability of the polymerization. The chain transfer constant of αMSD to MMA was approximately the same with αMSD to BA. The αMSD regulation mechanism was verified by NMR analysis, which was consistent with Yasummasa’s theory. In addition, the *R_p_* and *d_p_* of the acrylate latex were decreased as the increase in the dosage of αMSD, while the *N*_c_ of the emulsion was increased. Furthermore, the *PDI* was also affected when αMSD dosage was more than 1%. Finally, the decrease of the tensile properties of the resulting polyacrylate films was mainly due to the reduction in the molecular weights of synthesized polymers after the addition of αMSD.

## Figures and Tables

**Table 1:** Effects of αMSD concentration on average molecular mass, degree of polymerization, and monomer conversion. Note: CTA = chain transfer agent.

Run	CTA	(CTA/M ^1^)/%	M_n_/10^4^	Ð (M_w_/M_n_)	${\bar{X}}_{n}$ ^2^	Conversion/%
1	-	0.0	40.56	3.37	3612	100.0
2	αMSD	0.1	28.58	2.69	2545	97.8
3	αMSD	0.3	12.15	1.96	1082	97.6
4	αMSD	1.0	5.44	1.69	479	88.2
5	αMSD	3.0	1.79	1.61	122	47.3

^1^ M is the total weight of methyl methacrylate (MMA) and butyl acrylate (BA); ^2^ ${\bar{X}}_{n}$ is the number-average degree of polymerization.

**Table 2:** Formulas and values of *α*, *β*, *γ*, and *δ*.

Coefficients	α	β	γ	δ
Formulas	$\alpha =\frac{r_{2}}{1-r_{2}}$	$\beta =\frac{r_{1}}{1-r_{1}}$	$\gamma =\frac{1-r_{1}r_{2}}{\left(1-r_{1}\right)\left(1-r_{2}\right)}$	$\delta =\frac{1-r_{2}}{2-r_{1}-r_{2}}$
Values	0.52	−2.08	−0.56	−2.44

Note: *r*_1_ and *r*_2_ are reactivity ratios of MMA and BA (shown in Table 4), respectively.

**Table 3:** *Q–e* values of alpha methyl styrene (AMS), MMA, and BA.

Monomer	AMS	MMA	BA
*Q*	0.97	0.74	0.50
*e*	−0.81	0.40	1.06

**Table 4:** Reactivity ratios of AMS, MMA, and BA.

MMA/BA	MMA/AMS	AMS/BA
*r*_1_ = 1.93	*r*_3_ = 0.47	*r*_5_ = 0.43
*r*_2_ = 0.34	*r*_4_ = 0.49	*r*_6_ = 0.07

**Table 5:** Transfer coefficients (*C*_tr_) measured for αMSD.

Monomer	CTA	C_tr_	Reference
MMA	αMSD	0.62	39
BA	αMSD	0.47	39
MMA	αMSD	2.12	*Q*-*e*
BA	αMSD	14.28	*Q*-*e*

**Table 6:** Collection of latex particle size (*d*_p_) and its distribution (*PDI*) and particle number (*N*_c_) with αMSD at different concentrations.

Run	(CTA/M)/%	*d_p_* (nm)	PDI	*N*_c_ (10^17^ mL^−1^)
1	0.0	109.38	0.068	1.67
2	0.1	93.75	0.079	2.68
3	0.3	78.15	0.045	4.58
4	1.0	62.50	0.135	9.07
5	3.0	46.88	0.230	20.79

**Table 7:** Summary of Young’s modulus (*E*), tensile strength (*σ*_p_), and elongation at break (*ε*_b_) of films prepared using emulsion polymerization with αMSD as the CTA.

Run	(CTA/M)/%	*E* (MPa)	*σ*_p_ (MPa)	*ε*_b_ (%)
1	0.0	57.65	5.33	798
2	0.1	52.53	5.06	643
3	0.3	44.28	3.77	564
4	1.0	14.84	2.65	658
5	3.0	2.00	0.23	467
